# Uptake and Distribution of Soil Applied Zinc by *Citrus* Trees—Addressing Fertilizer Use Efficiency with ^68^Zn Labeling

**DOI:** 10.1371/journal.pone.0116903

**Published:** 2015-03-09

**Authors:** Franz Walter Rieger Hippler, Rodrigo Marcelli Boaretto, José Antônio Quaggio, Antonio Enedi Boaretto, Cassio Hamilton Abreu-Junior, Dirceu Mattos

**Affiliations:** 1 Centro de Citricultura Sylvio Moreira, Instituto Agronômico (IAC), Cordeirópolis, São Paulo, Brazil; 2 Centro de Solos e Recursos Ambientais, Instituto Agronômico (IAC), Campinas, São Paulo, Brazil; 3 Centro de Energia Nuclear na Agricultura (CENA/USP), Piracicaba, São Paulo, Brazil; Instituto Butantan, BRAZIL

## Abstract

The zinc (Zn) supply increases the fruit yield of *Citrus* trees that are grown, especially in the highly weathered soils of the tropics due to the inherently low nutrient availability in the soil solution. Leaf sprays containing micronutrients are commonly applied to orchards, even though the nutrient supply via soil could be of practical value. This study aimed to evaluate the effect of Zn fertilizers that are applied to the soil surface on absorption and partitioning of the nutrient by citrus trees. A greenhouse experiment was conducted with one-year-old sweet orange trees. The plants were grown in soils with different textures (18.1 or 64.4% clay) that received 1.8 g Zn per plant, in the form of either ZnO or ZnSO_4_ enriched with the stable isotope ^68^Zn. Zinc fertilization increased the availability of the nutrient in the soil and the content in the orange trees. Greater responses were obtained when ZnSO_4_ was applied to the sandy loam soil due to its lower specific metal adsorption compared to that of the clay soil. The trunk and branches accumulated the most fertilizer-derived Zn (Zn_dff_) and thus represent the major reserve organ for this nutrient in the plant. The trees recovered up to 4% of the applied Zn_dff_. Despite this relative low recovery, the Zn requirement of the trees was met with the selected treatment based on the total leaf nutrient content and increased Cu/Zn-SOD activity in the leaves. We conclude that the efficiency of Zn fertilizers depends on the fertilizer source and the soil texture, which must be taken into account by guidelines for fruit crop fertilization via soil, in substitution or complementation of traditional foliar sprays.

## Introduction

Zinc (Zn) deficiency is common in intensively managed citrus orchards in tropical soils and limits tree growth and fruit production [1]. This is mostly associated to the low nutrient content of the soil parent material and the high metal adsorption by the soil colloids. Therefore, Zn adsorption is maximal under high clay content and high pH. Adsorption under low soil pH (i.e. <5.5) is also important when iron and aluminum oxides predominate in the soil mineralogical fraction [2].

The acid soils are the main soil types in Brazil, which produces nearly 40% of the world’s orange juice. Other crops, such as sugar cane, coffee and rubber tree may also respond positively to Zn management under similar conditions [3,4].

Early studies have suggested that the application of Zn to acid soils with a sandy texture maintains the nutrient supply for a longer period than does Zn application to the leaves of plants [5]. However, questions still remain open regarding the efficacy of soil applied Zn in supplying citrus nutrient requirements for high yields when trees are grown on soils with a high Zn sorption capacity. This lack of information has led growers to continue to use foliar sprays as the main strategy for micronutrient fertilization of fruit trees in the tropics [6].

More recently, the efficiency of soil-applied Zn in complementing the leaf supply in sweet orange orchards was established in an Oxisol with a medium texture. A positive correlation was observed between the levels of micronutrients in the soil and those in the leaves when ZnSO_4_ was used as a source of soluble Zn [7]. Based on these results, a guideline was proposed for Zn availability as an auxiliary tool for fertilizer management recommendations in orchards [8]. Despite this latter contribution, no further information on the efficacy of Zn fertilizers that are less soluble in water is available for fruit crops. These types of fertilizers slowly release nutrients into the soil solution, thereby diminishing the adsorption of the metal to clay minerals, which may maintain the supply of the nutrient to the plant for a longer period, thus minimizing the risk of toxicity immediately after application as well as reducing the required frequency of application [9]. On the other hand, the efficacy of ZnO could be compared to that of more soluble fertilizer sources (as the sulfate) if applied to the acid soils [10] or mixed into the plow layer [11], which is of limited application, since soil disturbance would injury tree roots.

The isotope tracer method is a useful technique for studying nutrient mobility, absorption and distribution in plants. In the case of Zn, this technique can be applied by using either radioisotope (^65^Zn) or stable isotopes (^64^Zn, ^66^Zn, ^67^Zn, ^68^Zn, and ^70^Zn, which have respective natural abundances of 48.6%, 27.9%, 4.1%, 18.8%, and 0.6%). Only a few studies have employed this technique, including studies in pistachio and walnut [12], apple [13], peach [14], navy beans [15] and rice [16,17]. Furthermore, the selection of most appropriate methods of study is based on characteristics of every experiment, cost and precision of analytical detection of Zn isotopes by mass spectrometry.

In contrast to the isotopic approach, biochemical tests are indirect methods for assessing the nutritional status of a plant. The activity of superoxide dismutase (SOD) can be used as an indicator of uptake and content of copper (Cu), iron (Fe), manganese (Mn), and Zn by plants [18]. Thus, increases in the activity of Cu/Zn-SOD may be linearly correlated with the accumulation of Zn in the leaves [19]. The SOD is a key enzyme in the detoxification of the reactive oxygen species (ROS) that are formed in plants under stresses caused by nutritional disorders [20]. Moreover, the catalase (CAT) activity is altered in response to Zn and is inhibited by increasing the reactive oxygen species in plants [21].

In this context, limited information is available regarding the application of Zn fertilizers in tropical soils where citrus and other fruit trees are cultivated. In addition, the corresponding availability and adsorption of Zn and the associated nutritional status of plants have not been well studied. In the present study, we used the stable ^68^Zn isotope as a tracer to evaluate the efficiency of two sources of Zn with high and low solubilities in water applied to the soils of different textural classes. We also analyzed the plant growth, Zn partitioning, and the activity of SOD enzymes as an indicative of plant Zn status.

## Materials and Methods

### Plant materials and growth conditions

A greenhouse experiment was conducted using sweet orange [*Citrus sinensis* (L.) Osbeck cv. Tobias] grafted on ‘Sunki’ mandarin (*C*. *sunki* hort. ex Tan.) grown for 250 days in 20 dm^3^ pots containing a sandy loam or a clay type soil found in the main citrus growing areas of the tropics, which characteristics affect Zn availability to plants (Table 1).

**Table 1:** Selected soil characteristics.

Soil characteristics	Soil type	
	Sandy loam	Clay
O.M. (%)	2.2	3.7
pH in CaCl_2_	4.0	4.7
Base saturation (%)	23	58
CEC (mM_c_ dm^-3^)	68	125
P-resin (mg dm^-3^)	171	167
Exchangeable-K (mM_c_ dm^-3^)	4	6
Ca (mM_c_ dm^-3^)	21	32
Mg (mM_c_ dm^-3^)	26	22
B (mg dm^-3^)	0.8	3.0
Cu^a^ (mg dm^-3^)	0.9	2.8
Fe (mg dm^-3^)	113	24
Mn (mg dm^-3^)	3.8	9.0
Zn (mg dm^-3^)	0.4	1.9
Bulk density (g cm^-3^)	1.19	1.43
Total porosity (%)	50	35
Clay (%)	18.1	64.4
Silt (%)	5.1	16.6
Sand (%)	76.8	19.0
PMCF^b^	kaolinite	gibbsite and hematite

^a^Cu, Fe, Mn and Zn by DTPA-TEA extractor.

^b^Predominant mineral clay fraction by x-ray diffraction analysis.

Abbreviations: O.M. = organic matter; CEC = cation exchange capacity.

In both soils low pH was corrected with dolomitic lime to achieve a base saturation of 70% for optimum citrus growth [8]. During the experiment conduction, daily temperatures in the greenhouse were: minimum of 12°C, maximum of 37°C and mean of 23°C. The experimental treatments began when both of the soils achieved a pH (CaCl_2_) of approximately 5.2. The experiment was conducted in a completely randomized design with a 2x2 factorial design with two sources of Zn (ZnO and ZnSO_4_) and two soil textural classes (18.1 and 64.4% clay) replicated three times. There were no specific permits required for the described field studies. The field studies did not involve endangered or protected species.

The ^68^Zn-enriched isotope was acquired as ^68^ZnO (solid powder) from Isoflex USA (San Francisco, CA, USA), and presented the abundances: 99.34% ^68^Zn, 0.05% ^64^Zn, 0.08% ^66^Zn, 0.41% ^67^Zn, and 0.12% ^70^Zn. To prepare ^68^ZnSO_4_ [16], the ^68^ZnO was dissolved in 1.0 M H_2_SO_4_, gently stirred until the ^68^ZnO was completely dissolved and then diluted to make the volume up to 500 mL. The final concentration of ^68^ZnSO_4_ in the solution was 0.4 M of Zn and pH 4.4. The two sources were soluble (ZnSO_4_; 21% of Zn) or sparingly soluble (ZnO; 80% of Zn) in water [6].

The ^68^Zn-labeled fertilizer was applied when the plants presented two developed vegetative flushes with fully expanded and mature leaves. Three control plants were grown without Zn application to measure the Zn natural abundance. The Zn-containing fertilizers were applied once on the bare soil surface (0.102 m^2^), and were prepared either as a suspension (ZnO) or a solution (ZnSO_4_) in deionized water with a final volume of 500 mL per pot, which delivered 1.8 g Zn per plant.

Throughout the growth period, selected plant pots were weighted and irrigated as required to maintain approximately 70% of the soil field capacity. This latter was determined with 50 g of dry soil, placed in a funnel lined with a filter paper, and then saturated with 50 mL of water. Excess water in the soil sample was allowed to drain out. Soil field capacity was estimated by difference between volumes of water added and drained from the sample.

The fertilization for plant maintenance was conducted by applying 250 mL of solution each time with the following concentrations: 136.0 mg KH_2_PO_4_ L^-1^, 365.2 mg KNO_3_ L^-1^, 1,019.4 mg Ca(NO_3_)_2_ L^-1^, 114.7 mg CaCl_2_ L^-1^, 492.9 mg MgSO_4_.7H_2_O L^-1^, 2.9 mg H_3_BO_3_ L^-1^, 0.8 mg CuSO_4_ L^-1^, 0.25 mg NaMoO_4_.2H_2_O L^-1^, and 1.85 mg MnSO_4_.H_2_O L^-1^. From the planting to the harvesting of the plants, we conducted approximately 50 applications. Moreover, 100 mL of an iron solution (Fe-EDDHA, 5.0 mg L^-1^ Fe) was applied three times separately to the soil beneath each plant. These provided the following total amounts of nutrient in mg per plant: N (9,953), K (6,770), P (1,820), Ca (8,590), Mg (2,585), S (1,060), B (330), Cu (280), Fe (350), Mo (25), and Mn (460).

The plants were destructively harvested 150 days after ^68^Zn application, and plant tissues were separated into two parts: (i) old parts already grown at the time of fertilizer application and (ii) new parts developed after Zn fertilizers application. The flowers and fruits were also collected during the experimental period. All collected plant materials were washed with _d_H_2_O and dried to a constant weight. To determine Zn concentration in the plant tissues, the plant parts were ground to pass a 200-mesh sieve, digested with concentrated HNO_3_+HClO_4_ (2:1 v/v ratio) [22], and nutrient concentration was determined in the liquid extract by plasma emission spectrometry (ICP-OES, Perkin-Elmer 5100 PC, Norwalk, CT, USA). The Zn content of the soil samples was extracted with a DTPA-TEA solution at pH 7.3 and also determined by ICP-OES [23]. The soil analysis was conducted by measuring the sample volume with a scoop, which is a standard procedure, convenient and accurate, and the analytical result was expressed on a volume basis (mg dm^-3^)[24].

Adsorption curves were estimated to describe the adsorption capacity of Zn in the two soil types; a 1.0 g soil sample in 10 mL of a 10 mM CaCl_2_ solution containing Zn (0.5, 2.0, 8.0, 32.0, 128.0 and 512.0 mg L^-1^) as ZnNO_3_ was used. The tubes containing the samples were shaken for 48 h in a horizontal shaker at 160 rpm (25°C) and then centrifuged for 10 min at 905 × *g*. The supernatants were filtered, and the amount of Zn in the equilibrium solutions was quantified with an ICP-OES. The Langmuir isotherms were constructed using the sum of equilibrium concentration and the sum of adsorbed Zn [25]. This model best described the Zn adsorption capacity of the soils based on the experimental data when compared to the Freundlich model (data not shown).

### Isotopic determination

The ion intensities of all Zn isotopes (64, 66, 67, 68, and 70) were measured in a mass spectrometer coupled to a plasma source (ICP-MS) with an octapole reaction system, (mod. 7500ce, Agilent Hachioji-shi, Tokyo, Japan). In order to minimize polyatomic interferences (e.g., SO_2_ signal at mass 64), a robust plasma tuning was used to achieve the best possible long-term performance, low random background (<5 cps), enough sensivity (89Y > 60,000 cps/ppb), low metal oxides (CeO^+^/Ce^+^ < 0.7%) and doubly-charged ions (Ce^+2^/Ce^+^ < 1.0%). In order to optimize the ICP-MS operation parameters and calculate the mass discrimination factor, calibration standards, internal standards and certified reference materials (CRM) from SPEX Certiprep (Metuchen, NJ, USA) were used. Afterward, all Zn isotopes signals were corrected by the mass discrimination factor and the percentage of ^68^Zn was obtained by the following equation: [^68^Zn/(^64^Zn+^66^Zn+^67^Zn+^68^Zn+^70^Zn)] x 100. The measurements carried out by ICP-MS showed higher accuracy, with relative error lower than 0.95%, for all Zn isotopic ratios for the certified reference material (ISOT-Zn68 = 98,8%). The percentage of Zn in each plant part derived from the fertilizer source (%Zn_dff_) and the amount of Zn used by the plants (%AZn_f_) were determined via isotopic dilution (Equations 1 and 2). In addition, we calculated the sum of the estimated %AZn_f_ for all parts of the plant, the fertilizer use efficiency (FUE; Equation 3) and the partition of Zn_dff_ in the plant parts (PZn_dff_; Equation 4):

$${\text{\%Zn}}_{\text{dff}}\text{=(a-c)/(b-c)*100}$$ Equation 1

$${\text{\%AZn}}_{\text{f}}{\text{=\%Zn}}_{\text{dff}}{\text{*(Zn}}_{\text{p}}{\text{/Zn}}_{\text{f}}\text{)}$$ Equation 2

$${\text{FUE=(Zn}}_{\text{p}}{\text{/Zn}}_{\text{f}})*100$$ Equation 3

$${\text{PZn}}_{\text{dff}}{\text{=(Zn}}_{\text{dff}}\text{A/}\Sigma {\text{Zn}}_{\text{dff}}\text{ plant parts)*100}$$ Equation 4

where a is the ^68^Zn atom % in the fertilized plant material; b is the ^68^Zn atom % in the fertilizer; c is the ^68^Zn atom % of the non-fertilized plant material (control plants); Zn_p_ is the total amount of Zn in the plant material (mg plant^-1^); Zn_f_ is the total amount of ^68^Zn that was provided by the fertilizer (1.8 g plant^-1^); and Zn_dff_A is the Zn derived from the fertilizer in the plant part of the interest (mg plant^-1^).

### Enzyme activity

At the end of the experimental period, the SOD and CAT enzyme activities were determined in 1 g of leaves that were fully expanded and mature and that were grown after Zn application. Each sample was treated with 5.0 mL of 100 mM L-1 potassium phosphate buffer (pH 7.5) containing 3 mM L-1 dithiothreitol, 1 mM L-1 EDTA, and 4% (w/v) polyvinylpolypyrrolidone [26]. The extract was centrifuged at 5,590 × *g* for 35 min at 4°C, and the supernatant was used to determine protein levels and SOD activity. The total protein content was quantified according to Bradford [27] using bovine serum albumin (BSA) as a standard.

The superoxide dismutase activity was determined via 12% polyacrylamide gel electrophoresis (PAGE) [26]. Separation in non-denaturing gels was followed by incubation in the dark in a solution containing 50 mM L-1 potassium phosphate buffer (pH 7.8), 1.0 mM L-1 EDTA, 0.1 mM L^-1^ nitroblue tetrazolium, 0.05 mM L-1 riboflavin, and 0.3% tetramethylethylenediamine (v/v) for 30 min. After incubation, the reaction solution was removed, and the gels were rinsed with deionized water and exposed to light for several minutes to achieve photo-oxidation. The bands corresponding to the SOD activity did not undergo oxidation. Photo-oxidation was stopped via treatment with 7% acetic acid (v/v) for 15 min.

The following SOD isoforms were identified according to Azevedo et al. [28]: Cu/Zn-SOD when inactivated by KCN and H_2_O_2_; Fe-SOD when inactivated by H_2_O_2_ and resistant to KCN; and Mn-SOD when resistant to both compounds.

The catalase activity was determined via spectrophotometry [29]. The reaction was initiated via the addition of 25 μL of a plant extract containing 1.0 mL of 100 mM potassium phosphate buffer (pH 7.5) and 25 μL of H_2_O_2_ (30% solution) at 25°C. The enzyme activity was determined by following the decrease in the absorbance at 240 nm, which represents the decomposition of H_2_O_2_, for 1 min. The results were expressed in M min^-1^ mg^-1^ protein^-1^.

### Statistical analysis

Descriptive statistical analyses were applied to the data. A two-way ANOVA was performed to evaluate the effect of Zn-fertilizers and soil texture on the Zn in the soil and plants, the Zn_dff_ in the plants, and the catalase activity using the F test (PROC ANOVA, SAS version 9.2, SAS Institute, Cary, NC). When the interaction soil*source was significant, means were compared using the Tukey test at 5%. Pearson correlation was used to describe relationships between data sets (PROC CORR, SAS).

## Results

### Plant growth and zinc in soils and plants

The total dry matter (DM) production was higher for plants that received ZnSO_4_ than for those that received the less-soluble source of Zn (*p*<0.05). Furthermore, plant growth was 68% higher in the sandy loam soil (total DM = 484 g) than in the clay soil (total DM = 289 g; Table 2). The amount of young fruits that were produced at the end of the experimental period was proportional to the mass accumulation of the leaves (old + new) (R^2^ = 0.93, *p*<0.01). Within the same soil, the fruit production was 50% higher, on average, for the plants that received ZnSO_4_ compared to those that received ZnO (*p*<0.05, Table 2).

**Table 2:** Dry mass (DM) of plant parts of young orange trees 150 days after ZnSO_4_ and ZnO application in soils with sandy loam or clay texture.

Soil type	Zn source			F test^a^		
	ZnSO_4_	ZnO	Average	Soil	Source	Soil*Source
	**Young fruits** ^**b**^ **(g plant^-1^)**					
Sandy loam	63	31	47 A	**	*	ns
Clay	9	5	7 B			
Average	36 a	18 b				
	**Total DM (g plant^-1^)**					
Sandy loam	530	438	484 A	**	*	ns
Clay	295	282	289 B			
Average	413 a	360 b				

Soil type: the means (n = 3 or 6) followed by different uppercase letters within the columns are significantly different by the Tukey test (*p*<0.05). Zn sources: the means (n = 3 or 6) followed by different lowercase letters across paired columns are significantly different by the Tukey test (*p*<0.05).

^a^F test in the ANOVA of Soil vs. Source for each evaluated parameter. ns: not significant (*p*>0.05);

*p<0.05;

**p<0.01.

^b^Young fruits: 3–5 cm in diameter.

Although the level of available Zn in both of the soils was approximately 54 mg dm^-3^ (Table 3), the total concentrations in the different orange tree parts were higher when the source was ZnSO_4_, particularly in the sandy loam soil (Table 3). In addition, the Zn levels were highest in the roots of the orange trees. In the plants that were grown in the sandy soil that was treated with the sulfate source, the Zn content in the roots reached approximately 300 mg kg^-1^, approximately 10 times higher than that in the plants that were treated with the same fertilizer in the clay soil. Moreover, the Zn concentrations were higher in new branches and leaves than in the old parts of the plants. The Zn concentration in the flowers (17–33 mg kg^-1^) was proportional to that in leaves (R^2^ = 0.95, *p*<0.01).

**Table 3:** Concentration of Zn in the soil and plant parts of young orange trees 150 days after ZnSO_4_ or ZnO application in soils with sandy loam or clay texture.

Soil type	Zn source			F test^a^		
	ZnSO_4_	ZnO	Average	Soil	Source	Soil*Source
	**Soil (mg dm^-3^)**					
Sandy loam	58	51	54	ns	ns	ns
Clay	58	53	55			
Average	58	52				
	**Root (mg DW kg^-1^)**					
Sandy loam	306 Aa	20 Ab	163	*	*	*
Clay	33 Ba	18 Ab	26			
Average	170	19				
	**Trunk (mg DW kg^-1^)**					
Sandy loam	28 Aa	9 Ab	19	*	*	*
Clay	10 Ba	7 Aa	9			
Average	19	8				
	**Old branches (mg DW kg^-1^)**					
Sandy loam	44 Aa	9 Ab	27	**	**	**
Clay	14 Ba	8 Ab	11			
Average	29	9				
	**New branches (mg DW kg^-1^)**					
Sandy loam	61 Aa	10 Ab	36	**	**	**
Clay	12 Ba	10 Aa	11			
Average	37	10				
	**Old leaves (mg DW kg^-1^)**					
Sandy loam	21 Aa	10 Ab	16	**	*	*
Clay	12 Ba	10 Aa	11			
Average	17	10				
	**New leaves (mg DW kg^-1^)**					
Sandy loam	47 Aa	11 Ab	29	**	*	*
Clay	13 Ba	10 Aa	12			
Average	30	11				
	**Flowers (mg DW kg^-1^)**					
Sandy loam	33 Aa	21 Ab	27	**	**	**
Clay	20 Ba	17 Ba	19			
Average	27	19				
	**Young fruits^b^ (mg DW kg^-1^)**					
Sandy loam	13	6	10	*	ns	ns
Clay	11	11	11			
Average	12 a	9 b				

Soil type: the means (n = 3 or 6) followed by different uppercase letters within the columns are significantly different by the Tukey test (*p*<0.05). Zn sources: means (n = 3 or 6) followed by different lowercase letters across paired columns are significantly different by the Tukey test (*p*<0.05).

^a^F test in the ANOVA of Soil vs. Source for each evaluated parameter. ns: not significant (*p*>0.05);

*p<0.05;

**p<0.01.

^b^Young fruits: 3–5 cm in diameter.

### Zn adsorption capacity of soils

Maximum adsorption values, which are estimates of the maximum amount of Zn that these mineral surfaces can hold, were calculated for soil types based on the Langmuir isotherm [25]. The clay soil presented higher adsorption capacity of Zn (b_L_ = 1220 mg kg^-1^) than the sandy loam one (b_L_ = 790 mg kg^-1^). However, the binding energies between the metal and soil surface were similar for both, with an average value of 0.033 mg L^-1^ of Zn.

### Isotopic determination

The roots, stems, and branches accumulated more Zn_dff_ than did the leaves due to greater DM production. The Zn_dff_ content was much higher in younger leaves than in older leaves for all of the treatments (Table 4). However, this difference was less pronounced when the availability of Zn in the soil was likely higher in the short period following fertilizer application (e.g., ZnSO_4_ application in the sandy loam soil).

**Table 4:** Percentage of Zn derived from fertilizer (Zn_dff_) in young orange trees 150 days after the application of a Zn-labeled fertilizer (99.0 atom % excess ^68^Zn) in soils with a sandy loam or clay texture.

Soil type	Zn Source			F test^a^		
	ZnSO_4_	ZnO	Average	Soil	Source	Soil*Source
	**Root (% of Zn_dff_)**					
Sandy loam	99.7 Aa	40.1 Ab	69.9	**	**	**
Clay	42.6 Ba	7.6 Bb	25.1			
Average	71.2	23.9				
	**Trunk (% of Zn_dff_)**					
Sandy loam	96.3 Aa	25.2 Ab	60.8	*	**	**
Clay	41.2 Ba	11.2 Bb	26.2			
Average	68.8	18.2				
	**Old branches (% of Zn_dff_)**					
Sandy loam	97.5 Aa	22.1 Ab	59.8	**	**	**
Clay	41.4 Ba	4.3 Bb	22.9			
Average	69.5	13.2				
	**New branches (% of Zn_dff_)**					
Sandy loam	98.8 Aa	29.8 Ab	64.3	**	**	**
Clay	38.9 Ba	4.8 Bb	21.9			
Average	68.9	17.3				
	**Old leaves (% of Zn_dff_)**					
Sandy loam	47.7 Aa	0.1 Ab	23.9	**	**	**
Clay	5.2 Ba	0.1 Ab	2.7			
Average	26.5	0.1				
	**New leaves (% of Zn_dff_)**					
Sandy loam	97.3 Aa	15.8 Ab	56.6	**	**	**
Clay	33.3 Ba	6.5 Bb	19.9			
Average	65.3	11.2				
	**Flowers (% of Zn_dff_)**					
Sandy loam	60.0 Aa	13.7 Ab	36.9	**	**	*
Clay	26.8 Ba	2.7 Bb	14.8			
Average	43.4	8.2				
	**Young fruits** ^**b**^ **(% of Zn_dff_)**					
Sandy loam	83.7 Aa	21.8 Ab	52.8	**	**	**
Clay	41.6 Ba	9.0 Bb	25.3			
Average	62.7	15.4				

Soil type: the means (n = 3 or 6) followed by different uppercase letters within the columns are significantly different by the Tukey test (*p*<0.05). Zn sources: the means (n = 3 or 6) followed by different lowercase letters across paired columns are significantly different by the Tukey test (*p*<0.05).

^a^F test in the ANOVA of Soil vs. Source for each evaluated parameter. ns: not significant (*p*>0.05);

*p<0.05;

**p<0.01.

^b^Young fruits: 3–5 cm in diameter.

Among the fertilizer sources, the application of ZnSO_4_ to the sandy loam soil provided the greatest amount of this nutrient to the different parts of the plant, with the woody organs (stem and branches) and the new leaves exhibiting Zn_dff_>96% (Table 4). In contrast, the application of ZnO to the same soil resulted in Zn_dff_<42%, and this percentage was even lower in the clay soil, where Zn_dff_<12% (Table 4).

The Zn_dff_ content in the young leaves that grew after the fertilizer application was approximately 5 times higher than in old leaves (Table 5). The Zn_dff_ in young leaves, and flowers (R^2^ = 0.94), fruits (R^2^ = 0.96) among the different fertilizers and for both of the soils were well correlated (*p*<0.01).

**Table 5:** Zinc derived from fertilizer (Zn_dff_) in young orange trees 150 days after ^68^Zn application in soils with sandy loam or clay texture.

Soil type	Zn Source			F test^a^		
	ZnSO_4_	ZnO	Average	Soil	Source	Soil*Source
	**Roots (mg of Zn_dff_)**					
Sandy loam	65.32 Aa	1.10 Ab	33.21	*	*	*
Clay	2.50 Ba	0.19 Bb	1.35			
Average	33.91	0.65				
	**Trunk (mg of Zn_dff_)**					
Sandy loam	1.79 Aa	0.13 Ab	0.96	**	**	**
Clay	0.25 Ba	0.04 Bb	0.15			
Average	1.02	0.09				
	**Old branches (mg of Zn_dff_)**					
Sandy loam	2.08 Aa	0.09 Ab	1.09	**	**	**
Clay	0.14 Ba	0.01 Bb	0.08			
Average	1.11	0.04				
	**New branches (mg of Zn_dff_)**					
Sandy loam	1.28 Aa	0.08 Ab	0.68	**	**	**
Clay	0.07 Ba	<0.01 Bb	0.03			
Average	0.68	0.05				
	**Old leaves (mg of Zn_dff_)**					
Sandy loam	0.53 Aa	<0.01 Ab	0.26	**	*	*
Clay	0.02 Ba	<0.01 Aa	0.01			
Average	0.28	<0.01				
	**New leaves (mg of Zn_dff_)**					
Sandy loam	2.38 Aa	0.07 Ab	1.23	**	**	**
Clay	0.10 Ba	0.02 Bb	0.06			
Average	1.24	0.05				
	**Flowers (mg of Zn_dff_)**					
Sandy loam	0.02 Aa	<0.01 Ab	0.02	**	*	*
Clay	<0.01 Ba	<0.01 Aa	<0.01			
Average	0.01	<0.01				
	**Young fruits** ^**b**^ **(mg of Zn_dff_)**					
Sandy loam	0.72	0.04	0.38 A	**	*	ns
Clay	0.05	<0.01	0.02 B			
Average	0.39 a	0.02 b				

Soil type: the means (n = 3 or 6) followed by different uppercase letters within the columns are significantly different by the Tukey test (*p*<0.05). Zn sources: the means (n = 3 or 6) followed by different lowercase letters across paired columns are significantly different by the Tukey test (*p*<0.05).

^a^F test in the ANOVA of Soil vs. Source for each evaluated parameter. ns: not significant (*p*>0.05);

*p<0.05;

**p<0.01.

^b^Young fruits: 3–5 cm in diameter.

The partition of the Zn_dff_ (PZn_dff_) in the shoots of the orange trees was higher in the woody organs (stems and branches) than leaves, flowers and fruits, ranging from 58 to 73% of Zn_dff_, mainly due to the greater DM accumulation in the plants (Tables 2 and 6). However, in the case of the sandy loam soil that was treated with ZnSO_4_, in which a higher uptake of Zn by the plants was observed (Table 5), there was an increase in the partitioning of the Zn_dff_ to the leaves from 18% to approximately 30%, on average, corresponding to 2.9 mg Zn_dff_ plant^-1^. In the other treatments, only 0.1 mg plant^-1^ was accumulated. The use of ZnSO_4_ as the source of Zn led to the accumulation of 74.1 mg Zn_dff_ plant^-1^ in the sandy loam soil, representing the most efficient fertilization that was observed, at approximately 4%, compared to the other treatments (Table 6). The application of ZnO in the clay soil resulted in the lowest efficiency; only 0.01% of the total source material was absorbed by the plant.

**Table 6:** Partitioning of Zn derived from fertilizer (PZn_dff_) in young orange trees and the FUE of soil-applied Zn.

Plant parts	Sandy loam		Clay	
	ZnSO_4_	ZnO	ZnSO_4_	ZnO
***Aboveground***	---------------------------- % Zn_dff_ --------------------------			
**Flowers and young fruits**	8.3	11.7	10.4	9.1
**Leaves**	33.3	18.1	16.8	20.7
**Trunk + Branches**	58.4	70.2	72.8	70.3
	---------------------------- mg plant^-1^ --------------------------			
**Aboveground Zn recovered**	8.8	0.4	0.6	0.1
***Total plant***	--------------------------- % Zn_dff_ ----------------------------			
**New shoot**	6.3	9.6	9.2	11.7
**Old shoot**	6.7	11.1	21.0	19.9
**Root**	87.0	79.3	69.9	68.4
	---------------------------- mg plant^-1^ ---------------------------			
**Total Zn recovered**	74.1	1.5	3.1	0.3
	--------------------------------- % ---------------------------------			
**FUE** ^a^	4.15	0.12	0.16	0.01

^a^% of Zn recovered from the applied fertilizer.

### Enzymatic activities

**Fig 1:**
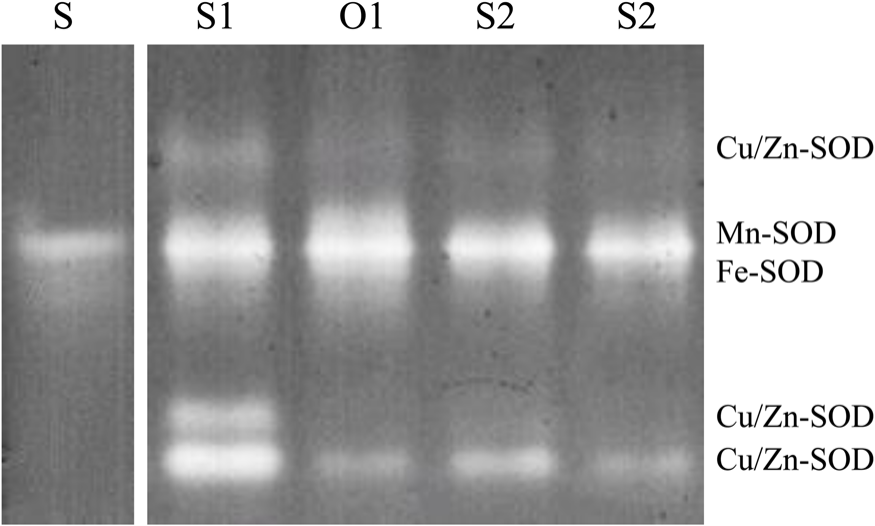
Superoxide dismutase (SOD) activity in the leaves of young orange trees 150 days after Zn application (enriched on ^68^Zn) in different soils. Legend: Bovine SOD standard (S), ZnSO_4_ (S1), and ZnO (O1) in the sandy loam soil; ZnSO_4_ (S2) and ZnO (O2) in the clay soil.

Following gel electrophoresis, three bands were identified for Cu/Zn-SOD, two for Mn-SOD and one for Fe-SOD. The Cu/Zn-SOD bands were the most highly expressed in the plants that were treated with ZnSO_4_ in the sandy loam soil (Fig. 1). The bands corresponding to Mn-SOD and Fe-SOD showed lower expression in the plant samples from the clay soil compared to those from the plants that were grown in the sandy loam soil. The CAT activity was not affected by the source of Zn and showed an average activity of 123 μM H_2_O_2_ min^-1^ mg^-1^ protein^-1^ (data not shown).

## Discussion

The solubility of ZnSO_4_ resulted in a higher efficiency in providing Zn to the plants, leading to an increase in the total DM (Table 2). This nutrient plays important roles in protein synthesis and the attenuation of damages caused by ROS [2,21], which likely explains the total and fruit DM yields that was observed in our study. This later effect occurred in response of the improved nutritional status of leaves [2,30] by taken up Zn_dff_ (Tables 2 and 5).

Also relevant to the differences in the DM production of the plants that were grown in the sandy loam and clay soils (Table 2) is the fact that citrus trees display a good adaptability to different soil conditions in order to maintain high yields. However, the higher apparent density and porosity in the clay soils are disadvantageous for subsurface aeration, consequently limiting the root and shoot growth [31]. Although this study attempted to provide an equivalent water supply to each experimental pot, anaerobic microsites may occur at a higher frequency in the clay soil (total porosity *P* = 35%) than in sandy soil (*P* = 50%), which explains the differences in DM production that were observed

The flower DM did not differ among the treatments. Nevertheless, the Zn levels ranged from 10 to 47 mg kg^-1^ in this organ (*p*<0.01) (Table 3), suggesting that growing flowers positively responded to the nutritional status of the plant. The literature does not cite specific Zn-regulated mechanisms that directly affect the quality of the flower, as have been demonstrated for other nutrients, such as calcium and boron [32,33]. Therefore, the observed higher fruit yield observed is likely due to the increased leaf area and consequently greater capacity for carbon allocation that flowering requires [34,35]. Similarly, the increased iron concentrations in the leaves during the flowering season of orange trees are associated with increased CO_2_ assimilation by citrus in orchards that are established in calcareous soils [29]. Furthermore, in this latter study, the Zn concentrations in the leaves and flowers were poorly correlated due to the low observed levels (15 mg kg^-1^ in the leaves and 12 mg kg^-1^ in the flowers) compared to those levels that were found in our study, in which the plants that were grown in the sandy loam soil were treated with the ZnSO_4_ (47 mg kg^-1^ in the leaves and 33 mg kg^-1^ in the flowers) (Table 3).

The high content of Zn in the roots of plants (>300 mg kg^-1^) is associated with the greater compartmentalization and lower metal transport to the shoots, which results in the resistance or tolerance of plants to excessive concentrations in order to maintain homeostasis [37]. Accordingly, the percentage of Zn_dff_ (>99%) and the Zn_dff_ content (65 mg) were much higher in the roots than in the other organs (stems, leaves, flowers, and fruits) (Tables 4 and 5). Furthermore, the Zn accumulation in roots could supply this nutrient to the shoots, even at small quantities, for longer periods [5] compared to the accumulation in the leaves, developed after foliar applications [36].

The higher %Zn_dff_ value in the new leaves compared to that in the older leaves (Table 4) suggests that younger tissues act as a stronger Zn sink, even considering that the Zn_dff_ was diluted by the nutrient content that was present in the older leaves prior to isotope labeling. The flowers displayed an increase in the Zn_dff_ content in parallel with Zn accumulation in the new leaves. Under the conditions of this experiment, the uptake of Zn in the young leaves did not limit the intake of Zn in the flowers, in contrast to previous observations of the distribution of Fe in orchards of orange trees [33]. This difference may be explained by their relative demand in the rapidly growing pattern of leaves vs. flowers, which alters the transport and distribution of nutrients in plants [37].

The higher solubility of ZnSO_4_ compared to that of ZnO led to higher total Zn levels and, consequently, the greater contributions of Zn (Zn_dff_) in the orange trees (Tables 3 and 5). When the former fertilizer was applied to the sandy soil, the leaf content in the plants was 43 mg kg^-1^ (Table 3), corresponding to a 97% Zn_dff_ in the young leaves (Table 4). Levels in the range of 25 to 50 mg kg^-1^ of Zn in the leaves of citrus plants are sufficient to ensure vigorous growth [8]. However, visual diagnosis of these leaves should also be performed to correctly interpret the nutritional status of the plants because Zn deficiency is associated with reduced auxin production and subsequent limited tissue growth [38], which causes leaf blade deformation and alters the concentration of this element when expressed on the basis of the dry weight of the tissue [2]. The average area of the new leaves in the plants that were treated with Zn was greater (35 cm^2^ leaf^-1^) than that of the leaves in the untreated plants (23 cm^2^ leaf^-1^).

In this experiment, the plants were evaluated for during one cycle of vegetative growth following fertilizer application. Besides the decreased availability of Zn that was applied to the soil because of the partition of the metal in fractions that are bound to soluble forms, complexed with organic colloids, or linked to Mn and Fe oxides [39], the residual effects of Zn could explain the Zn accumulation in the subsequent flush of plant growth [40]. In addition, the efficacy of Zn fertilizers varies with incorporation of the fertilizer into the soil [2]. In our study the fertilizers were applied to the surface, which might have limited deeper incorporation of those into the soil. Similarly, the ZnO efficacy also was lower compared to more soluble Zn sources, when the fertilizers were not mixed into the soil of wheat [11] and maize [41].

In all of the treatments, the old leaves accumulated the lowest Zn_dff_, with the exception of the flowers, which may have been due in part to the lower DM yield of these leaves in contrast to that of the other developing organs, such as the stems and branches (Table 5). Thus, the old leaves are not the preferred target organs of root-absorbed Zn. Nutrient accumulation in old parts of plants is expected to occur only when the availability of Zn in the soil solution is sufficiently high [40]. Furthermore, under conditions of low nutrient supply, remobilization can occur from older and mature tissues to those that are undergoing development [37]. Thus, in addition to the new leaves, the stems and branches are important reserve organs for Zn, as a high content of this element was observed in the aboveground parts of the plants (Table 6), similarly to verified in other crops like as coffee and beans [40,42]. However, there is also no strong evidence regarding plant capacity for nutrient remobilization through the phloem among different plant species, analogous as reported for boron with species that accumulated polyols [43].

Despite variations in the concentrations of applied Zn, there was no difference in the levels of other nutrients, including P, Mn, and Cu (data not shown), in the leaves; an excess of these nutrients could negatively affect the absorption and distribution of Zn in plants [44]. For instance, with high P supply under a limiting Zn condition, the physiological availability of Zn in plant tissue decreases [45]. For other metals, such as Fe, Mn or Cu, the high levels of these cause a certain competition with Zn for absorption sites [44].

The fertilizer use efficiency (FUE) is defined as the amount of Zn that is recovered by the plant as a proportion of the amount of Zn supplied by the fertilizer. The FUE was higher for ZnSO_4_, particularly when applied to the sandy loam soil due to the greater solubility of this source. The pH value of both of the soils was approximately 5.2. Consequently, the difference in the FUE was dependent on the physical and mineralogical features of those soils, which determine their adsorptive capacity (b_L_) (clay = 1220 mg kg^-1^ and sandy loam = 790 mg kg^-1^). The clay soil exhibited a higher b_L_ for Zn [2] suggesting that the implementation of Zn recommendations should at least consider the textural differences that occur in the orchards of the main citrus production regions.

Of the total Zn supply, the proportion of plant uptake ranged from 0.01% to 4.15% for ZnO in the clay soil and ZnSO_4_ in the sandy loam soil, respectively (Table 6). These values were lower than those that were obtained by Boaretto et al. [34], who found that approximately 6% of the ZnSO_4_ (0.7 mg L^-1 65^Zn) that was applied to the leaves was absorbed by orange trees. However, despite this greater recovery of the nutrient following foliar application, the absorption of ^65^Zn occurred primarily during the first several days after fertilization, after which no significant absorption was detected. Under the conditions of this experiment, the time between sampling and the ^68^Zn fertilization of the plants was 150 days. This time period may have been insufficient to allow for more significant root absorption, which would have increased the fertilizer efficiency, particularly for the ZnO in the clay soil. This result would be supported by a previous study in which the soil application of ZnSO_4_ in a medium-texture Oxisol [7] demonstrated increased foliar levels of field-grown trees only in the second year after fertilization.

Moreover, when Zn was applied to the soil, this mineral is distributed to all of the parts of the plant, in contrast to the application of fertilizer to the leaf, after which less than 1% of the ^65^Zn is redistributed to the other parts of the plant [34]. For this reason, in citrus trees and other tropical fruit trees, the foliar application of Zn is recommended only during spring and summer when new vegetative flushes grow [8].

The SOD activity assay was effective in determining the presence of biologically active Zn in the plants [21], since an increase of the enzyme activity occurred for the three bands corresponding to the Cu/Zn-SOD with the treatment that resulted in the highest Zn content in the leaves (47 mg kg^-1^) (S1; Fig. 1). Total SOD activity was detected despite Zn content was lower with other treatments (11 mg kg^-1^) (S2, O1 and O2; Fig. 1). On the other hand, under severe Zn deficiency, SOD activity increases relative to plant micronutrient bioavailability resulted directly by a decrease of functional Zn and/or indirectly by an increase of Fe and Mn, which cause an increase of ROS production [19].

Although an increase in Cu/Zn-SOD activity was observed, there was no increase in CAT activity. Thus, the increase in the metal level in the plant tissue (Table 2) did not cause oxidative stress.

Thus, based on our findings, the establishment of an adequate strategy for soil application of the micronutrient for citrus and other fruit crops, in substitution to the traditional foliar sprays, should consider the effect of the soil texture and the quality of the fertilizer source. However, make it necessary to note that in the case of fertirrigated orchards, given the thermodynamic characteristics of the soil solution under drippers other issues that define the fertilizer efficiency characteristic for this system should also be considered.

## Conclusions

The Zn fertilizer use efficiency, based on the recovery rate in the plants, was as high as 4% when a water-soluble Zn source was used in a sandy loam soil, but it decreased to only 0.1% when a less soluble ZnO source was used on the clay soil with the highest Zn sorption capacity. Under these conditions, the accumulation of Zn_dff_ occurred mainly in the roots and woody organs, which may be important reserve organs for this nutrient. Despite the relatively low fertilizer efficiency, the nutrient requirement of the citrus plants was likely satisfied, as verified by the increased Zn contents in the leaves, the higher DM production, and the increased SOD activity.
